# Characterization of the complete chloroplast genome of *Arisaema erubescens* (Wall.) Schott, a traditional Chinese medicinal herb

**DOI:** 10.1080/23802359.2020.1797577

**Published:** 2020-08-12

**Authors:** Yan Zhang, Xiuzhi Guo, Binbin Yan

**Affiliations:** National Resource Center for Chinese Meteria Medica, China Academy of Chinese Medical Sciences, Beijing, PR China

**Keywords:** *Arisaema erubescens*, chloroplast genome phylogenetic relationship

## Abstract

*Arisaema erubescens* is a well-known medicinal plant in China. In this study, we sequenced the complete chloroplast (cp) genome sequence of *A. erubescens* to investigate its phylogenetic relationship in the family Araceae. The cp genome was 167,607 bp in length, consisting of a pair of inverted repeats (IRa and IRb: 26,193 bp) separated by a large single-copy region (LSC: 93,660 bp) and a small single-copy region (SSC: 21,561 bp). The GC content of whole cp genome is 35.3%. *De novo* assembly and annotation showed the presence of 114 unique genes with 80 protein-coding genes, 30 tRNA genes, and four rRNA genes. Phylogenetic analysis indicated that *A. erubesc*ens was closely related to *A. franchetianum*, and the genus *Arisaema* was sister to the genus *Pinellia*.

*Arisaema erubescens* (Wall.) Schott is a perennial herb of the Araceae family, which is widely distributed in south China and Southeast Asian countries. It is used medically as an important antidote to treat several biological disorders (Li et al. 2010). With the discovery of the great medicinal value of *A. erubescens* and the increasing demand of the market, the wild resources of *A. erubescens* are decreasing nowadays. It is necessary to develop genomic resources for *A. erubescens* to provide intragenic information for its utilization. Chloroplast genomes are valuable sources of genetic markers for phylogenetic analyses, genetic diversity evaluation, and plant molecular identification (Dong et al. 2018; Sun et al. 2020). In this study, the complete chloroplast genome of *A. erubescens* was assembled to provide genomic and genetic resources for further research, and the phylogeny of family Araceae was conducted to reveal the interspecific relationships.

Fresh samples of *A. erubescens* were collected from Hongjiang county, Hunan province, China (27°24′38′′N, 110°25′19″E). Voucher specimen was deposited at the herbarium of Institute of Chinese Materia Medica (CMMI), China Academy of Chinese Medical Sciences with the specimen voucher number is 431281LY0254. Total genomic DNA was isolated using the CTAB method (Doyle and Doyle 1987). The library with 350 bp insertion size fragments was constructed and sequenced using the Illumina HiSeq platform. *De novo* assembly was conducted using GetOrganelle pipeline (Jin et al. 2018). The annotation was performed using Plann (Huang and Cronk 2015), with annotated complete chloroplast (cp) genome from *A. ringens* (NC044118) selected as reference. The annotated complete chloroplast cp genome of *A. erubescens* was submitted to the GenBank under the accession number MT676834.

The complete cp genome of *A. erubescens* was a circular DNA molecule of 167,607 bp in length, containing a large single-copy region (LSC) of 93,660 bp, a small single-copy region (SSC) of 21,561 bp, and a pair of inverted repeat (IR) regions of 26,193 bp. The overall GC-content of the cp genome was 35.3%. The IR regions had a higher GC content (41.4%) than that of the LSC (33.2%) and SSC (29.5%) regions. The cp genome contained 114 genes, including 80 protein-coding genes, 30 tRNA genes, and four rRNA genes. In these genes, six tRNA genes (*trnK-UUU*, *trnG-UCC*, *trnL-UAA*, *trnV-UAC*, *trnI-GAU*, and *trnA-UGC*) and nine protein-coding genes (*atpF*, *ndhA*, *ndhB*, *petB, petD*, *rps16*, *rpl16*, *rpl2*, and *rpoC1*) contain one intron, while two genes (*ycf3* and *clpP*) have two introns.

To investigate the phylogenetic relationships between *A. erubescens* and other related species in family Araceae, 20 complete cp genomes were downloaded from GenBank to construct a phylogenetic maximum-likelihood (ML) tree using RAxML (Stamatakis 2014), under the GTR + G model with 1000 rapid bootstrap replicates. *Lemna minor* (DQ400350) was taken as an outgroup. The phylogenetic tree showed that all 19 species forming a monophyletic group supported by a 100% bootstrap value. *A. erubescens* was inferred phylogenetically closest to *A. franchetianum*, and the genus *Arisaema* was sister to the genus *Pinellia* (Figure 1). The complete cp genome of *A. erubescens* provided a lot of genetic information for species conservation and identification as well as the phylogenetic studies of family Araceae.

**Figure 1.**
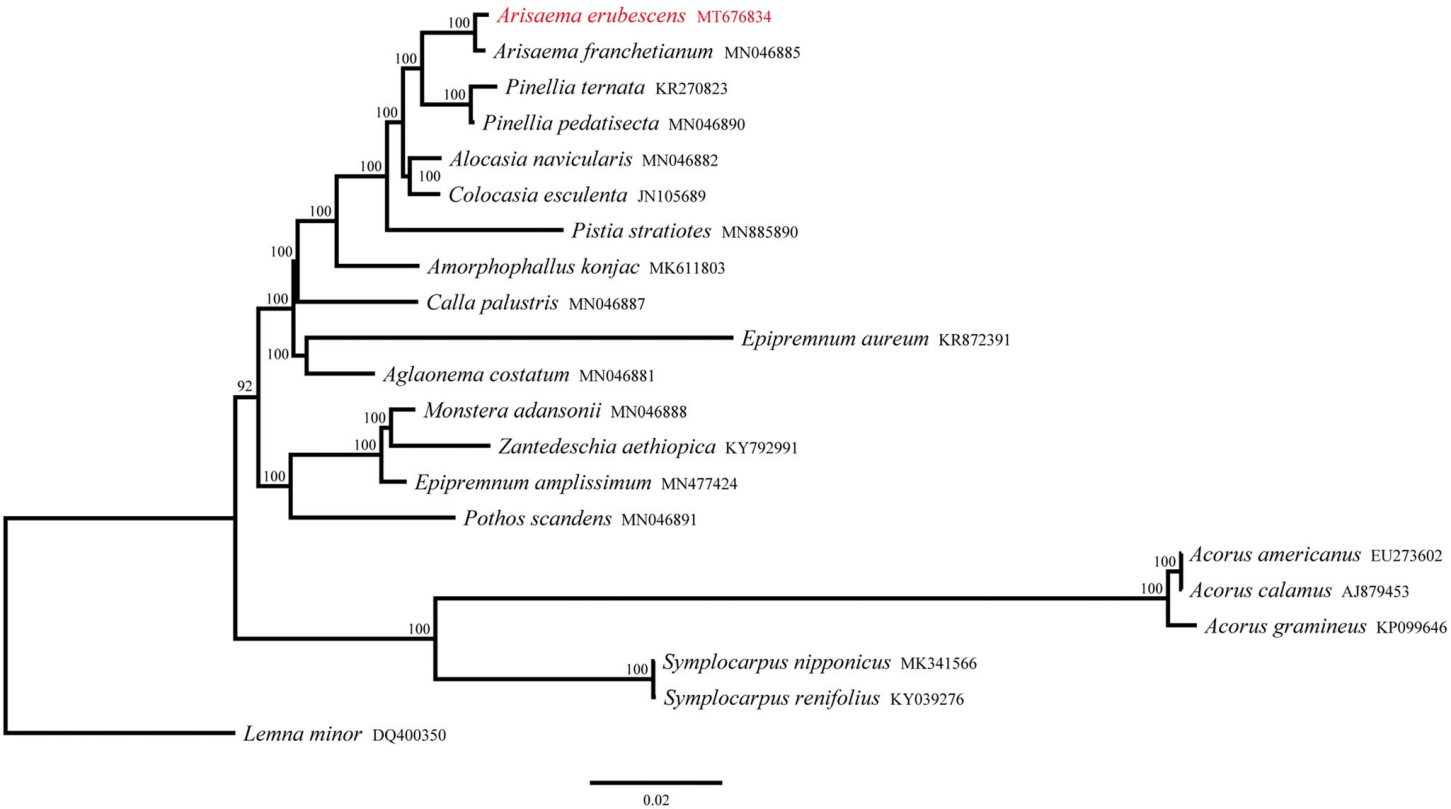
Phylogenetic tree reconstruction of 21 taxa using maximum-likelihood (ML) methods based on 77 genes in the chloroplast genome sequences. ML bootstrap support value presented at each node.

## Data Availability

The data that support the findings of this study are openly available in GenBank of NCBI https://www.ncbi.nlm.nih.gov/, reference number MT676834.
